# The impact of violence on women’s health. The present as a reflection of the past: A qualitative study

**DOI:** 10.1371/journal.pone.0273973

**Published:** 2022-09-09

**Authors:** Rebeca García-Montes, Sandra Fares-Medina, Isabel Diaz-Caro, Inmaculada Corral-Liria, Soledad García-Gómez-Heras

**Affiliations:** 1 Student International Doctoral School, Health Sciences Faculty, King Juan Carlos University, Alcorcón (Madrid), Spain; 2 Nurse University Hospital Severo Ochoa, Leganés (Madrid), Spain; 3 Nursing and Stomatology Department, King Juan Carlos University, Alcorcón (Madrid), Spain; 4 Basic Health Science Department, King Juan Carlos University, Alcorcón (Madrid), Spain; Flinders University School of Medicine: Flinders University Medicine, AUSTRALIA

## Abstract

The objective of the study is to analyze the impact of violence on women’s health and the feelings generated during the period of exposure to violence. This is a qualitative study with an interpretative phenomenological design in which 16 women participated—4 through interviews and 12 through stories. The data obtained were analyzed using the Colaizzi approach. The results were structured into 3 themes according to how the women interpreted their experiences. The themes were physical consequences: visible traces; psychological consequences: stormy days and sunny days; and social consequences: from loneliness to a new world. In conclusion, the women in this study considered all those (very diverse) physical pathologies to be important. They encompassed a series of psychological disorders that lasted over time, causing considerable suffering and complicating the participants’ ability to relate to the rest of society, especially men. Similarly, the participants identified a series of positive consequences when they left the traumatic situation empowered after overcoming gender-based violence.

## Introduction

Gender-based violence (GBV), or violence against women, as the WHO has designated it, is one of the most important social health problems in Spain, and the WHO has considered it a public health problem. GBV is defined by many as any act of physical or psychological violence, including assaults on sexual freedom, threats, coercion or arbitrary deprivation of liberty, in both the public and private spheres, by someone who is or has been linked to the woman through an emotional relationship, even if the perpetrator and the woman do not live together [1–3].

In Europe, the prevalence of these events exceeds 25%. Likewise, in Spain, in 2020, more than 150,000 complaints were filed for gender violence, 9,004 electronic surveillance devices were activated and almost 36,000 restraining orders were enacted. With these data, gender violence has become one of the most important social problems in Spain, whit the deaths of 45 women, without any previous disease attributable to GBV [4, 5].

Regarding the consequences, 78.2% of women who experienced any type of violence reported that it affected their physical or mental health, without specifying the type of effects. In addition, women identify a great variety of feelings—impotence, sadness, fear, anguish, shame, guilt and aggression—when they recall the abuse that they suffered [5].

The impact of violence on women’s health is significant. The main physical and sexual and/or reproductive consequences of violence are chronic pain syndromes, fibromyalgia, irritable bowel syndrome and other gastrointestinal disorders, lacerations and abrasions of the skin, eye damage, disability, infertility, complications with pregnancy and/or abortion, sexually transmitted diseases, unwanted pregnancy and even death. The main psychological effects are anxiety, and abuse of power and control, insomnia, chronic fatigue, chronic disorders, such as post-traumatic stress or depression; and negative feelings [6–11].

Consequently, investigating the impact of these consequences on women who have suffered GBV, is essential since very little research is currently available, and adequately training psychosocial professionals would be highly beneficial. By pointing out the needs and/or shortcomings that women who have experienced this type of violence identify, it is possible to reinforce and deepen these aspects, and therefore improve and implement training that benefits women who are victims of abuse. The objective of this study is to analyze the impact of violence on the health of women and to describe the feelings generated by exposure to violence.

## Materials and methods

### Design

This is a qualitative phenomenological interpretive study based on the principles of Heidegger [12–16].

The sample was chosen through intentional and snowball sampling of women who had suffered GBV. Access to the sample was gained through the mediation of the Miriadas of Huelva Association, via email, which, since its creation in 2002, has been dedicated to caring for women who are victims of abuse and their minor children by providing emergency care by telephone 24 hours per day, accompanying and monitoring them in the process of leaving violent situations and offering weekly training and therapeutic workshops [17]. A sample of 16 women was obtained, and data saturation was achieved in the course of the analysis [18].

The inclusion criteria a) were majority age (18 years), according to Royal Decree-Law 33/1978 of November 16 in Spain; b) residence in Spain, where the study was carried out; c) being separated from their aggressors and having had no contact with them for 1 or more years, because despite the absence of a specific time in which gender violence is overcome, a year or more separated from the partner can be indicative of a firm commitment to overcome the abuse; d) actively working or seeking work, since this is already part of the “plan” initiated by a woman to restart her life; e) and volunteering to participate in the study.

Women were excluded if talking about their experience of GBV generated physical and/or psychological suffering or if they lived in short- and medium-stay residential institutions, such as shelters, because women who still live in these types of housing do not do so independently, and these women will need to be independent in the future.

### Data collection

The data were collected from 4 of the participants through semi-structured interviews carried out during personal meetings lasting 1,5 hours, which were recorded; the other 12 participants preferred to participate by sharing their stories, by postal mail using the same questions that were used in the interviews. The data were collected on the laptop of the investigators. The questions asked are included in Table 1.

**Table 1:** Questions asked to informants during the process of lived violence.

1. Questions asked to informants about the process of violence experienced and their feelings about it.	At what point did you realize that you were being a victim of gender-based violence? How did she recognize him? What feelings did this identification produce in you?
2. Questions asked to informants about the end of the violence experienced and their feelings about it.	What did you do to stop the abuse by your ex-partner? What was the reason or trigger that made you decide to get out of that situation? What feelings did he give you?
3. Questions asked to informants about the role their family played in the process of violence they experienced	What was the attitude of your family and trusted people in the process of overcoming gender violence? What would you change regarding the performance of these people?
4. Questions asked to informants about resources and social tools during the process of violence they experienced.	What resources and social tools did you find available to you? How did you access them? What difficulties did you encounter in accessing them? Which ones do you think were most and least useful? Are there any social resources that you missed?
5. Questions asked to informants about personal and psychological coping and coping tools and mechanisms during the process of violence they experienced.	What personal and psychological tools and mechanisms for overcoming and coping have you used throughout this process? What tools have you been acquiring?
6. Questions asked to informants about the physical, psychological and social consequences during the process of violence and the feelings experienced.	Currently, what physical, psychological and social consequences of the situation of gender violence that you experienced do you consider that you present? What feelings and emotions does it produce to remember the process of detachment from his former partner, and consequently, from the situation of abuse?
7. Questions asked to the informants about some aspect that has not been mentioned during the process of violence experienced.	Is there any question that has not been asked and that you think is interesting to comment on? If yes, what is it?

### Data analysis

The Colaizzi approach described by Northall [19] was selected for the data analysis, since it is one of the accepted data analysis methods used in qualitative interpretive research in the field of Nursing [20, 21]. This approach consists of 7 points:

1. Reading the women’s descriptions.
2. Obtaining relevant statements for each description and coding them.
3. Providing a detailed explanation of the meaning of each relevant statement to formulate meanings and facilitate further coding.
4. Organizing the formulated meanings into thematic groups.
5. Comparing the thematic groups with the original statements to validate them and resolve discrepancies.
6. Providing an exhaustive description of the phenomenon with the results obtained in the previous steps.
7. Consulting with the participants to validate the original data.

The 4 principles of bioethics governed this study. The principle of beneficence was upheld by using the data extracted from the women’s interviews to help women who are currently victims of abuse and by caring for and respecting the participants of the study. The principle of nonmaleficence was upheld by ensuring that none of the participants was intentionally harmed. The principle of autonomy was upheld by accepting and respecting the decisions of the women, who voluntarily participated in the study, were able to leave it at any time and signed their informed consent (IC) [22]; furthermore, the study adhered to Organic Law 3/2018 of December 5 regarding the protection of personal data and the guarantee of digital rights [23]. Finally, the principle of justice guided the study, since the goal of applying the results obtained is to improve the care provided by professionals who treat women who have survived gender violence based on real experiences. In addition, the study was approved by the Research Ethics Committee of the Rey Juan Carlos University of Madrid [24], and was governed by the Consolidated criteria for reporting qualitative research (COREQ) [25].

The quality of the results of the study was controlled according to the quality principles of Guba and Lincoln descrited by Forero et al. [26] Credibility is present when data are collected through interviews and stories; confirmability is present when the characteristics of the women are described and their contributions are transcribed verbatim; and transferability is present when the findings obtained can be transferred to other similar studies [27].

## Results

The final sample of the study consisted of 14 women, aged between 21 and 64 years, who had a relationship with their aggressors for between 1,5 and 40 years, and did not have contact with him for between 1 and 15 years. The considerable variability in both the ages of the participants and the lengths of time in the relationship and separated from the aggressor up to the day of the study increases the richness of the results in terms of varied experiences. (See Table 2).

**Table 2:** Sociodemographic characteristics of the participants.

Participant code	Age	Years suffering from GBV	Years without contact with the aggressor
1	32	3	3
2	49	19	3
3	36	3	13
4	39	10	3.5
5	55	20	15
6	53	40	2
7	31	5	9
8	39	14	3
9	21	1.5	4
10	21	1.5	3
11	57	35	2
12	64	20	7
13	30	4	2
14	24	2	1

Three main themes emerged from the data: physical consequences: visible traces; psychological consequences: stormy days and sunny days; and social consequences: from loneliness to a new world. (See Table 3).

**Table 3:** Results: Themes and subthemes.

**Theme: Physical Consequences: visible traces**	
**Subthemes**	**Relevant Issues of Subthemes**
Very Diverses	Effects on the digestive system
	Effects on the mouth
	Effects on the genitals
	Effects on the musculoskeletal system
Sleep disorders	Nightmares
**Theme: Psychological consequences: stormy days and sunny days**	
**Subthemes**	**Relevant Issues of Subthemes**
Negatives	Anxiety
	Anguish
	Suffering
Continuous intrusion of memories	An unforgettable experience
Not feel capable of comfortably talking openly	
Feeling sadness, shame or pity	
Confusing when the recognized Themselves as victims of GBV	
Positives	Personal growth
	Feeling liberation, relief and strength
	Confidence in themselves
	Feeling of feminism from the abuse
	Feelings of doubt and hopelessness
	Feelings of security, pride and improvement
**Theme: Social consequences: from loneliness to a new world**	
**Subthemes**	**Relevant Issues of Subthemes**
Negatives	Loneliness
	Blaming the woman
	Social indifference
	Vulnerability
	Tied to the aggressor
Positives	New network of people
	Feeling grateful
	Feeling happy
	Reinserting into work life
	Independence
Difficulties	Developing a sharp rejection of the male gender
	Feeling fear
	Feeling hate

### Physical consequences: *Visible traces*

The physical consequences were considered the most visible since they could be observed by the rest of society. Consequently, the women granted them relatively high importance since they were a physical burden that they saw and felt every day and that, on many occasions, followed them throughout their lives. These consequences were very diverse, affecting various areas of the body, including the digestive system, the mouth, the genitals and the musculoskeletal system.

- *“Physical consequences: whiplash” (6)*
- *“Physical consequence: fibromyalgia” (1)*
- *“I still have physical consequence: a tooth.” (7)*

One of the most frequently recurring health problems was sleep disorders, either as a bodily response to the psychological stress the women suffered or as a result of nightmares about their aggressor. In either case, sleep was considered an important vital aspect since allowed the body and mind to rest and renewed the women’s energy on a day-to-day basis, and its absence added a daily burden to their ongoing struggle.

- *“I do not sleep well, […] and many things are coming out, and the nightmares.” (3)*
- *“[…] and my body reacts by sweating a lot, sleeping little, whit diarrea, vomiting, panic attacks and anxiety.” (8)*

### Physical consequences: *Stormy days and sunny days*

Regarding psychological consequences, the women identified them as mostly negative and related their traumatic events to the psychological problems they experienced. Many of the participants reported having disorders such as anxiety, which they felt was a result of the abuse since it was something they could not control that paralyzed them, made them remember the anguish they felt during episodes in which they did not know what to do, and the suffering that all this caused.

- *“I have anxiety, specifically; I suffer from everything.” (5)*
- *“I felt control, insecurity, loneliness and a desire to end the situation, but I did not feel capable.” (8)*
- *“I felt bad for putting up with it, but I was aware that I had an emotional dependence.” (7)*

Another consequences of the abuse was the continuous intrusion of memories prompted by day-to-day circumstances. The majority of the participants agreed that their experience could never be forgotten; in fact, some admitted that they still did not feel capable of talking openly about the abuse since they still felt sadness, shame or pity for themselves and even felt confused when the recognized themselves as victims of GBV.

- *“I am still afraid to this day, and sometimes I feel like I want the same things that I had with him with my current partner” (14)*
- *“I still keep many things reserved.” (3)*
- *“Remembering causes me anger, sadness, but at the same time, relief.” (11)*
- *“Then, also when you realize that yes, that you are just one more, that when they talk about who knows how many victims of GBV abuse, well, you realize that you are one more, that you had suffered that, it kicks you a thousand times, because that is not just you. That happens to others.” (14)*
- *“The feeling that reminds me of it is one of sorrow—sorrow for having allowed it and not having finished with all this sooner” (7)*
- *“[…] but I never believed myself to be the victim of anything, but rather I felt that we were both in a loop in which we were self-destructing, and I did not feel like a victim of anything.” (14)*

On the other hand, the personal growth that some of the participants experienced when they emerged from this undesirable life situation stronger, with improved self-esteem and proof to themselves of what they were capable of doing was inevitably something to highlight. These women did not hesitate to express that they felt liberated and relieved, that they had been reborn stronger and with greater confidence in themselves.

- *“I do not sleep well, […] and many things are coming out, and the nightmares.” (4)*
- *“I felt relieved and with the strength to give myself a chance without him.” (7)*
- *“I am stronger, more aware of what gender violence is and what I never want to experience again.” (8)*
- *“I, alone, I throw myself forward; I don’t need anyone, and I can do it.” (14)*

It was possible to see that the participants recognized an incipient feeling of feminism from the abuse, which could be interpreted as a desire or need for union between equals to defend themselves, decrease their feelings of doubt and hopelessness and increase feelings of security, pride and improvement.

- *“Let’s see; I have a hypersensitivity to any topic related to women, from macho language to any subtle commentary of the micromachismo type. I am hypersensitive.” (14)*
- *“I don’t think I’m over it yet.” (2)*
- *“I will never forget what I experienced.” (1)*
- *“I have forgiven him; I feel nothing for him.” (5)*
- *“So to say, ‘Well, you did great; that is, you thought of yourself from within the self-destructive loop you were in’ because when I look at it from a distance, I say, ‘So, good for me, because the truth is that I caught it in time.’ That is what they told me—that I caught it in time.” (14)*

### Social consequences: *From loneliness to a new world*

The social repercussions for the participants were important, since one of the most common feelings during violence was loneliness. They were greatly affected by society’s attitudes towards them, especially the approach of blaming the woman, which the participants themselves even started to internalize, and social indifference, which made them even more vulnerable.

- *“I was alone.” (2)*
- *“Their behavior was ambivalent; they support you, but at the same time, they distrust you.” (10)*
- *“I felt disbelief, guilt.” (10)*
- *“I started running even more, and he followed me out and tried to hit me, he almost caught me… and I didn’t know what to do or anything. I started shouting, ‘Please help!’, and I was crying a lot, and nobody, nobody came out to see what was happening, why I was screaming… nothing.” (14)*
- *“I am not afraid of failing, but I am afraid of letting them do the same to me again.” (7)*

On the other hand, psychologically, some of the women came out of this experience stronger, with a new network of people they could trust and from whom they received support and affection, and feeling grateful and happy for this. Similarly, some women who felt completely tied to the aggressor by their contempt became reinserted into working life, returning to their previous jobs or training for new ones, thereby becoming independent.

- *“I started to work, to relate to people and to lose my fear little by little, although that is never completely gone.” (3)*
- *“I am good, thanks to so many years at Miriadas; they made me a woman again, stronger, more positive.” (9)*
- *“I am completely or almost recovered; I am happy, and I have inner peace within myself (…).” (4)*
- *“I felt bad for putting up with it, but I was aware that I had an emotional dependence.” (7)*
- *“I felt invalidated.” (2)*
- *“Once I got out, I realized that I was alone and that I did not have to depend on him as I thought I had.” (12)*

Regarding the way the women related others after GBV, some of them confessed having difficulties due to their distrust and the negative interpretations of accepted social gestures. For this reason, they developed a sharp rejection of the male gender caused by the fear they suffered and the anger they felt toward their aggressor.

- *“Currently, I do not have any physical consequences, but it has caused me a lot of insecurity and fear of starting a new relationship.” (7)*
- *“I still have a general fear of the male sex, his clothing, his way of walking…” (13)*
- *“Fear that he might be capable of doing something very bad to his own daughters.” (6)*
- *“I felt hatred.” (9)*

## Discussion

Similar to the findings of the present study, Hegarty [28], the Panamerican Health Organization [29], Aguilar-Palacio [30] and Bolaños [31] in theirs studies on the impact of GBV on women’s health, revealed that pathologies such as gastrointestinal disorders, specifically irritable colon, were related to sexual assaults; fibromyalgia, chronic pain, sleeping problems and gynecological diseases were the most frequently described physical conditions, and most of these conditions were mentioned by the participants of this study, who were able to identify them clearly. On the other hand, these studies described a significant number of traumatic injuries, which were not part of the experience of the participants in the present study; which may suggest that these injuries were implied in the physical aggressions that they experienced or that the participants in the present study did not experience traumatic injuries.

Tiruye [32] and Karakurt [33] spoke of accentuated psychopathological symptoms, which they indicated were the most frequently reported impacts for women who experienced GBV. Among these symptoms were anxiety, which included panic attacks in some reports which the women in this study describing profuse sweating, nervousness, stress or worry about small things that should not matter in additions to phobias, which the women in this study described as fear of everything related to the male gender. Notably, some feelings, specifically guilt and shame, were identified as consequences of abuse, and the women in this study identified these feelings both during the abuse phase and as they overcame it.

Authors such as Lutgendorf [34] and Sugg [35] did not include the social consequences and their impact on women’s health; instead, they focused only on physical, mental and/or psychological, sexual and reproductive health. In contrast, the present study observed the ways that abuse involves changes at the social level that affect women’s health and should not be ignored.

The results of the present study mostly support the existing literature, but some consequences that had not been mentioned before were discovered, along with the feelings that women experienced in each stage, which brings us closer to understanding the experiences of women who have survived GBV. These revelations can help social and mental professionals ensure that they are meeting most of the needs that women have when overcoming abuse. However, the lack of research on the impact of gender violence on the health of women indicates that further efforts are needed to expand knowledge about the experiences of these women.

One of the greatest limitations of this study, which may explain the limited research on this topic, is difficult access to participants, as not very many women feel prepared to share this experience or are able to share without being harmed, in addition to the fact that socially, they are very protected by the associations where they find extensive biopsychosocial help.

## Conclusion

The women in this study considered all of these varied physical pathologies important and described a series of psychological disorders that lasted over time, burdening them with enormous suffering that made it difficult to relate to the rest of society, especially men. Similarly, they identified a series of positive outcomes when they left this traumatic situation empowered after overcoming GBV.

These women also emphasized that their way of relating to other people and their entire social environment had changed considerably as a result of their experience with GBV.
